# Antibacterial efficacy of ultrasonically activated probiotic endodontic irrigant against *Enterococcus faecalis* biofilm: an *in-vitro* study

**DOI:** 10.1186/s12903-025-06212-x

**Published:** 2025-05-26

**Authors:** Mai Sayed Hanafy, Noha Mohamed Kamal, Hebatallah Atef Fathallah

**Affiliations:** 1 Endodontic Department, Faculty of Dentistry, Suez University, Suez, Egypt; 2 Botany Department, Faculty of Women for Arts, Science, and Education, Ain Shams University, Heliopolis, Egypt; 3 Endodontic Department, Faculty of Oral and Dental Medicine, Nahda University in Beni Suef, Beni Suef, Egypt

**Keywords:** Probiotics, *Lactobacillus plantarum*, *Enterococcus faecalis* biofilm, Ultrasonic-activated irrigation, Antibacterial activity

## Abstract

**Background:**

*Enterococcus faecalis* (*E. faecalis*) is the most frequently retrieved microorganism from teeth with failed endodontic treatment. Sodium hypochlorite (NaOCl) irrigant still poses some drawbacks, such as its cytotoxic effect and reduced effectiveness when applied at lower concentrations. Root canal disinfection by probiotics may yield positive outcomes due to their proven antibacterial and anti-inflammatory abilities. This research was intended to assess the antibacterial efficacy of a probiotic irrigant after ultrasonic activation against *E. faecalis* in a tooth model.

**Methods:**

Teeth specimens were infected with *E. faecalis* biofilm and then randomly divided into five groups according to the final flush irrigation protocol used; PRO for probiotic irrigant, PRO + for activated probiotic irrigant, NaOCl for NaOCl irrigant, NaOCl + for activated NaOCl irrigant, and saline for saline irrigation. Activation of the irrigant was done for 1 min using an Ultra X ultrasonic tip. By counting the colony-forming units per milliliter, the antibacterial activity was quantitatively evaluated for each group pre- and post-irrigation application; then, the bacterial load reduction percentages were calculated accordingly. The one-way ANOVA was conducted to compare the mean values of all variables, followed by the post-hoc Tukey test to make group comparisons with a significance level set at *p* < 0.05.

**Results:**

All experimental groups exerted antibacterial activity against *E. faecalis* with a reduction in the mean CFUs/mL values and an increase in the mean bacterial load reduction percentages. The lowest mean post-irrigation CFUs/mL values were observed in the NaOCl + group, followed by NaOCl, PRO + , PRO, and saline groups respectively. Statistically significant differences were observed among all groups, except for the NaOCl and PRO + groups which did not exhibit any statistically significant difference.

**Conclusion:**

Ultrasonically activated probiotic irrigant revealed an antibacterial effect similar to the conventional NaOCl and can be effectively used to fight against *E. faecalis* biofilm.

## Background

Endodontic treatment failure may still occur even with the most meticulous care taken during root canal procedures [1]. The primary factor contributing to this failure is mainly the survival of harmful pathogens within the obturated root canals leading to persistent pulpal infection [2]. The *Enterococcus faecalis (E. faecalis)* is the most frequently retrieved microorganism from persistent infections in teeth with failed endodontic treatment [3, 4]. E. *faecalis* can grow inside the root canals as a single organism without relying on other microbes and can form a biofilm [5]. It also can survive in extreme environmental conditions, tolerating starvation for a prolonged period of time [4, 5]. Consequently, *E. faecalis* is regarded as one of the most unmanageable intracanal microbes, as it resists the antimicrobial activity of many endodontic irrigants and intracanal medicaments [2, 4].

Various approaches to combating *E. faecalis* were investigated by utilizing different irrigants [6]. Although sodium hypochlorite (NaOCl) remains a popular root canal irrigant for *E. faecalis* biofilm removal due to its antibacterial properties and ability to dissolve organic tissue [7]. Still, it poses some drawbacks, such as its cytotoxic effect, potential irritation for the periapical tissues, and reduced effectiveness against some microorganisms when applied at lower concentrations [7]. Therefore, there is a rising interest in creating more biologically accepted approaches to improve the effectiveness of *E. faecalis* elimination.

The traditional endodontic assumptions often insist on the total elimination of every microorganism in the root canal system, regardless of its specific characteristics or potential pathogenicity [8]. This is based on the belief that any presence of bacteria could lead to the failure of endodontic therapy [9]. However, new research is beginning to question this approach suggesting that it may be unnecessary to eradicate all microorganisms. Instead, the focus is shifting towards controlling the microbial population by keeping it in a dynamic equilibrium rather than achieving complete sterility [10]. Consequently, this will manage the surrounding environment, prevent reinfection, and promote healing. Probiotics can properly serve this concept while improving microbial resilience when used in the oral cavity [11].

Probiotics, which are living microorganisms, generally offer health benefits when included in dietary products [12]. They are actively used now as a biological medication for the management of many chronic gastric diseases and other different medical conditions [12]. Probiotics can inhibit the formation of pathogenic biofilm by competing for nutrients with disease-causing microorganisms thus preventing their growth [13]. The concept of probiotics has already been implemented in the field of dentistry for the control and prevention of dental carious lesions and the management of periodontal diseases, oral halitosis, and oral candidiasis [14]. Root canal disinfection by probiotics may also yield positive outcomes due to their proven antibacterial and anti-inflammatory abilities [15]. However, more studies are needed to validate their applicability in endodontic therapy.

*Lactobacillus* bacterial strains are one of the most commonly used probiotics in dentistry due to their strong wide-range antimicrobial properties that render them an effective regimen for both treating and preventing infections [15]. *Lactobacillus* probiotic strains have already been reported to have an antibacterial action against *E. faecalis* as evaluated in previous studies [16–18].

For better root canal system disinfection, the efficiency of disinfecting irrigating solutions can be maximized within the root canal space using different irrigation activation techniques [19]. These techniques include activation through manual dynamic agitation, lasers, sonic and ultrasonic devices [19]. Ultrasonic-activated irrigation can agitate root canal irrigants leading to the removal of dentin debris, pulp tissues, and resistant bacteria from the root canal by the shear stresses produced by acoustic streaming of the irrigant [19].

To our knowledge, no research addressed the effect of ultrasonic activation for probiotics when used as irrigants on their antibacterial activity against intracanal-resistant pathogens. So, this research was intended to assess the antibacterial efficacy of a probiotic irrigant after ultrasonic activation against *E. faecalis* biofilm in a tooth model. The present study was based on the null hypothesis suggesting that the use of probiotics as an irrigant would not cause a significant change in the bacterial load of the *E. faecalis* biofilm.

## Methods

Ethical committee approval (SUEZIRB-MED/810241) was obtained from the Ethical Committee of Suez University in Egypt, and the study was conducted in accordance with the Declaration of Helsinki. All subjects who donated their teeth for the current study and/or their legal guardians voluntarily agreed, and informed consent was obtained.

### Probiotic and pathogenic strains selection

The *Lactobacillus plantarum* (*L. plantarum*) ATCC 14917 probiotic strain was purchased (Himedia Laboratories Pvt. Ltd, India) and used for this research. The standardized microbiological protocol (ATCC guidelines) was followed to extract the probiotics, which was done under strict aseptic conditions. *E. faecalis* ATCC 29212 was chosen as the endodontic pathogen (American Type Culture Collection, Virginia, USA).

### Microbial strains storage and maintenance

Strains used in this study were preserved by mixing 24 h old cultures grown in de Man, Rogosa, and Sharpe (MRS) broth with sterile cryoprotectant (glycerol) and storing the mixture in cryogenic vials at −80°C. To activate the frozen bacteria, it was thawed at room temperature, used to inoculate MRS broth, and incubated under anaerobic conditions for 24–48 h at 37 °C. Culture viability was tested by subculturing on MRS agar and confirming colony morphology, observing Gram-stained slides and examining other characteristics, e.g., catalase activity, in case of *L. plantarum*, and subculturing on Blood agar and confirming colony morphology, Gram stain reaction, and blood hemolysis, in case of *E. faecalis*.

### Probiotic cultivation and supernatant preparation

The *L. plantarum* was inoculated in a tube containing De Man, Rogosa, and Sharpe (MRS) broth (Neogen®, Lansing, MI, USA) and incubated in Heraeus B5042 incubator under anaerobic conditions for 48 h. The concentration of bacteria was OD_600_ = 0.6. An aliquot (1 mL) of this culture was used to inoculate a flask containing 100 mL MRS broth and then incubated under anaerobic conditions for 72 h at 37 °C.

The cell-free supernatant (CFS) was obtained by centrifugation at 10,000 rpm for 10 min using Megafuge16 centrifuge (Thermo Fisher Scientific Inc., USA), sterilized by syringe filter size of 0.2 microns, and then lyophilized via a freeze dryer (Martin Christ Alpha 1–4 LD, GmbH, Germany). The freeze-dried CFS was then stored at −20°C [20].

### Probiotic Minimum Inhibitory Concentration (MIC) assessment

The lyophilized probiotic CFS was suspended in sterile water to obtain a 300 mg/mL probiotic solution. The MIC of the probiotic irrigant and its antibacterial activity were assessed against *E. faecalis* using two methods: agar well diffusion assay and broth microdilution method.

#### Agar well diffusion assay

*E. faecalis* was inoculated on blood agar (Himedia® Laboratories Pvt. Ltd, India) and incubated at 37 °C for 24 h under anaerobic conditions. A cell suspension was prepared and adjusted to the concentration of half McFarland standard (1.5 × 10^8^ CFUs/mL) using a spectrophotometer (Spectronic 21D; Milton Roy Co., Rochester, NY). A blood agar plate was inoculated with an aliquot of cell suspension using a sterile cotton swab. After that, a 6 mm cork borer was used to make wells on the agar plate. Using sterile distilled water, CFS with different dilutions were prepared with concentrations of 0.1 to 300 mg/mL. An aliquot (200 μl) of each dilution was loaded to each separate well, while pure sterile water was regarded as a negative control. Then, the plates were incubated in an incubator at 37 °C for 24 h. Zone of inhibition (ZOI) was observed and measured in millimeters (mm). The lowest concentration of CFS which showed ZOI was regarded as MIC.

#### Broth microdilution method

MIC of *L. plantarum* CFS against *E. faecalis* was also evaluated using the broth microdilution technique following the CLSI (Clinical Laboratory Standards Institute) guidelines. Two-fold serial dilutions of CFS (from 300 to 0.1 mg/mL) were prepared using Mueller–Hinton broth (Himedia® Laboratories Pvt. Ltd, India), and 100 µL was loaded in flat-bottom wells of a microtiter plate. A culture (24-h old) of *E. faecalis* in Mueller–Hinton broth was prepared and used to inoculate the microtiter plate wells to give a final 5 × 10^5^ CFUs/mL concentration. After inoculation, the plate was incubated under anaerobic conditions at 37 °C for 24 h. After incubation, optical density (OD) was measured at 630 nm via a microplate reader (Lonza ELx808™ Incubating Absorbance Plate Reader). The lowest concentration of CFS, which inhibited visible growth of *E. faecalis,* was regarded as MIC; and the value obtained using this method was compared with the MIC obtained by agar well diffusion technique.

### The MIC determination for the L. plantarum probiotic against E. faecalis

**Fig. 1:**
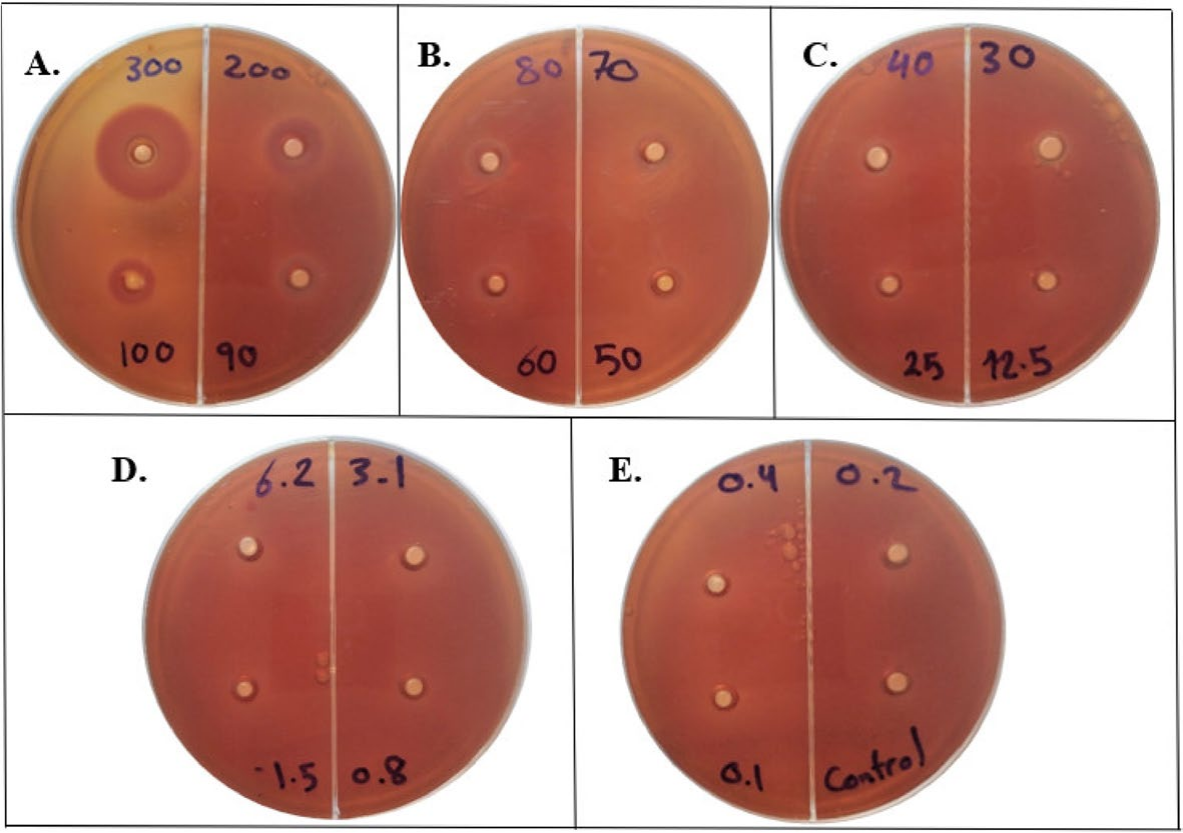
The MIC of *L. plantarum* probiotic against *E. faecalis* determined via agar well diffusion assay. **A** Displays the ZOI for probiotic concentrations of 300 mg/mL, 200 mg/mL, 100 mg/mL, and 90 mg/mL. **B** Shows the ZOI for probiotic concentrations of 80 mg/mL, 70 mg/mL, 60 mg/mL, and 50 mg/mL. **C** Depicts the ZOI for probiotic concentrations of 40 mg/mL, 30 mg/mL, and 25 mg/mL, with no ZOI observed at 12.5 mg/mL. The MIC was found to be 25 mg/mL with a 7.25 mm ZOI. **D** & **E** Indicate no ZOI for probiotic concentrations of 6.2 mg/mL, 3.1 mg/mL, 1.5 mg/mL, 0.8 mg/mL, 0.4 mg/mL, 0.2 mg/mL, 0.1 mg/mL, and the control

The MIC of *L. plantarum* probiotic irrigant, measuring antibacterial activity, was done by testing gradient concentrations (300 to 0.1 mg/mL) using the agar well diffusion assay. MIC was found to be 25 mg/mL. Results revealed significant differences in mean ZOI diameter between 300, 200, 100, 50, and 25 mg/mL concentrations (Table 1 and Fig. 1).

**Table 1:** Comparison of zone of inhibition diameter in millimeters for different concentrations of *L. plantarum* probiotic against *E. faecalis* via agar well diffusion assay

Probiotic Concentration (mg/mL)	ZOI diameter (mm) Mean ± SD
300	24.25 ± 1.26^A^
200	17.00 ± 1.15^B^
100	12.50 ± 1.29^C^
90	11.25 ± 1.26^C^
80	9.37 ± 0.48^D^
70	9.25 ± 0.29^D^
60	8.87 ± 0.48^D^
50	8.25 ± 0.50^D^
40	7.62 ± 0.2^E^
30	7.50 ± 0.58^E^
25 (MIC)	7.25 ± 0.29^E^
12.5	ND
6.2	ND
3.1	ND
1.5	ND
0.8	ND
0.4	ND
0.2	ND
0.1	ND
Control	ND
*p-value*	< 0.001*

Data presented are the means of quadruplicates ± standard deviation. Means with the same capital superscript have no significant difference at *p* < 0.05. *ZOI* Zone of inhibition, *ND* Not detected, *Significant

### The MIC validation for L. plantarum probiotic against E. faecalis

The MIC of *L. plantarum* probiotic irrigant was determined at 25 mg/mL concentration using the broth microdilution method which was similar to that obtained by the agar well diffusion assay (Table 2).

**Table 2:** The MIC validation of *L. plantarum* probiotic using the broth microdilution method

Probiotic Concentration (mg/mL)	Visible Growth (Y/N)
300	N
200	N
100	N
50	N
25 (MIC)	N
12.5	Y
6.2	Y
3.1	Y
1.5	Y
0.8	Y
0.4	Y
0.2	Y
0.1	Y
Control	Y

*Y* Yes (growth), *N* No (no growth)

### Preparation of specimens

Sample size calculations were performed using open-source software (G*Power 3.1, Universidad Düsseldorf, Germany). A significance threshold of 5%, an effect size of 0.35, and a power of 80% were used to plan the sample size. To compensate for any laboratory errors, it was raised from the initial calculation of five per group to seven. So, the total sample size needed to assess the antibacterial efficacy of different final root canal irrigation protocols = the number of groups × the number of specimens per group = 5 × 7 = 35 teeth.

A total of 35 freshly extracted, permanent, caries-free, single-rooted, single-canalled human teeth were selected for this study. Teeth were obtained from patients routinely extracting their teeth in the Maxillofacial Surgery Clinic, Faculty of Dentistry, Suez University, due to periodontal involvement.

A surgical operating microscope was used to examine the teeth to exclude any teeth that had cracks or other flaws. Radiographs were obtained at various angles to assess the morphology of the roots. After being carefully cleaned under running water, teeth were submerged in 5.25% NaOCl (Clorox, Egypt) for 30 min to detach the root surface’s soft tissues. An ultrasonic scaler (Varios 550, NSK, Nakanishi, Japan) was used to remove any calculus or soft tissue remnants from the root surface. Then, the teeth were stored in 9% saline (Sodium Chloride, Al Mottahedoon Pharma, Egypt) till instrumentation.

Decoronation of the teeth was performed to standardize the teeth lengths at 16 mm. An access cavity was created in each tooth, and a size #10 K-file (Mani Inc., Tokyo, Japan) was used to establish canal patency. A size #15 K file was inserted until the tip was visible just past the apex under magnification and then 1 mm was deducted from the file length to determine the working length (WL). Mechanical preparation was performed for all root canals to the WL using NiTi ProTaper Next rotary system (Dentsply Sirona Maillefer, Switzerland) up to size X3 (0.30 tip size/7% taper). One milliliter of NaOCl (2.6%) was dispensed between files during instrumentation. A 19% ethylene-diamine-tetra-acetic acid (EDTA) gel (MD-Chelcream, Meta Biomed Co Ltd, Korea) was applied to each rotary file before being introduced inside the root canal.

Following instrumentation, the root canal was flushed with 3 mL of 17% EDTA solution (EDTA Plus, Essential Dental Systems, New Jersey, USA) for 5 min, and then 3 mL of 2.6% NaOCl to remove smear layer. To inactivate NaOCl, 5% sodium thiosulfate (PIOCHEM, Egypt) was used as a final rinse. Then, the canals were dried with absorbent paper points (Meta Dental Co. Ltd., Korea).

A nail varnish was applied in a double layer to the root’s outer surfaces and light-cured composite resin (Filtek Supreme Z250, 3M ESPE, USA) was used to seal the teeth’s apical foramina to prevent bacterial contamination. Each specimen was placed in an Eppendorf and then autoclaved for 30 min at 121 °C and was handled thereafter under strict aseptic measures. To ensure sterility, extra teeth specimens, excluded from the total sample size, were prepared in the same way and autoclaved, then used to inoculate blood agar plates that were incubated at 37 °C for 48 h.

### Contamination of specimens with E. faecalis biofilm

*E. faecalis* ATCC 29212 was inoculated on blood agar and incubated at 37 °C for 24 h under anaerobic conditions. Colonies were suspended in a tube containing 5 mL of brain heart infusion (BHI) broth medium (Himedia® Laboratories Pvt. Ltd, India), and OD was adjusted to a concentration of 1 McFarland (3.0 × 10^8^ CFUs/mL) using Milton Roy Spectronic 21D spectrophotometer. A sterile insulin syringe was used to inoculate each root canal with 10 μl of *E. faecalis* suspension, and the canals were then incubated for 21 days at 37 °C in aseptic anaerobic conditions. To make sure the bacteria were viable, the inoculation was carried out every 72 h with new BHI broth [21, 22]. The viability of *E. faecalis* and the formation of biofilms were confirmed in the root canals by inoculating *E. faecalis* in the wells of a microtiter plate containing Trypticase soy broth supplemented with 1% glucose and viewing the developed biofilm via staining with crystal violet [23, 24].

### Irrigation protocols

The prepared teeth specimens were randomly divided into five main groups (7 teeth each) according to the final flush irrigation protocol used:

- PRO Group: Probiotic irrigant (300 mg/mL) was used.
- PRO + Group: Probiotic irrigant (300 mg/mL) was used and then activated for 1 min.
- NaOCl Group: A positive control group where NaOCl (2.6%) irrigant was used.
- NaOCl + Group: NaOCl (2.6%) irrigant was used and then activated for 1 min.
- Saline Group: A negative control group where saline irrigation was done.

The probiotic irrigant was prepared by mixing the lyophilized CFS of the probiotic with sterile distilled water to obtain a 300 mg/mL concentration irrigant based on the findings of the agar well diffusion technique conducted for probiotic MIC determination.

All irrigants were dispensed passively into the root canal in 3 mL volume for 2 min using a syringe with a side-vented needle (0.3 × 25, ENDO-TOP®, Cerkamed, Poland) which was positioned 1 mm short of the WL. Activation of irrigant was done using an ultrasonic tip (flexible X Silver tip) (size #20, 2% taper) attached to an ultrasonic activator device (UltraX, Eighteeth, Changzhou Sifary Medical Technology Co., Ltd, China). The tip was positioned 1 mm short of the WL and activated for one minute at 45 kHz (maximum power) in an up-and-down motion. Five milliliters of distilled water (Pharmapack, Pharmaceutical Industries, Egypt) were then used to rinse the root canals to flush out any remnants of the irrigant and then dried with sterile paper points.

### Randomization and blinding

The irrigation protocols were assigned codes, grouped, and sealed in opaque envelopes. A random sequence was created using computer software (http://www.random.org/). The laboratory operator, data collector, and statistician were blinded to the irrigation protocol employed in each group; however, the operator was aware of it during the procedure.

### Assessment of the antibacterial activity of different final irrigation protocols

By counting the number of *E. faecalis* colony-forming units per milliliter (CFUs/mL), the antibacterial activity was quantitatively evaluated for each group pre- and post-irrigation application; then, the bacterial load reduction percentages were calculated accordingly.

After 21 days of *E. faecalis* incubation within the root canals, the bacterial count was recorded before any intervention. To obtain pre-irrigation samples (S1) from the canals of every tooth, a sterile size #40 paper point was inserted till reaching the apical foramen, moved circumferentially along the walls of the canal, and left for 5 min. The paper points were then removed from the root canals and put in 2 mL-sealed cryogenic vials containing 1 mL BHI broth.

After applying the final irrigation protocol, an immediate second post-irrigation bacterial sample (S2) was taken to record the count of *E. faecalis* by placing a sterile size #40 paper point into each root canal for 5 min. Then, the point was kept in 2 mL sealed cryogenic vials containing 1 mL BHI broth to prevent contamination.

Each paper point sample (pre- and post-irrigation) was vortexed for three consecutive 15-s intervals, followed by serial tenfold dilutions (1:10 to 1:1000000). Using an automatic micropipette, 50 μL of each dilution was placed in Perti dishes over which liquified BHI agar was then poured and thoroughly mixed. The experiment was carried out in quadruplicates. The following mathematical formula was used to determine the percentages of bacterial load reduction between S1 and S2 [25]:

$$\text{Bacterial Load Reduction Percentage }= \frac{\text{SI}-\text{S}2}{\text{S}1}\times 100$$

where S1 is the pre-irrigation count (CFUs/mL) and S2 is the post-irrigation count (CFUs/mL).

The data was statistically analyzed utilizing the SPSS (Version 27—IBM Corp, NY, USA). The CFUs/mL values were assessed for normality using the Shapiro–Wilk and Kolmogorov Smirnov tests and were found to be normally distributed. A significance level of p < 0.05 was set to determine statistical differences between the tested groups. The one-way ANOVA was conducted to compare the mean values of all variables, followed by the post-hoc Tukey test to make group comparisons.

## Results

### Interpretation for comparison of CFUs/mL and bacterial load reduction percentage before and after different irrigation protocols

**Fig. 2:**
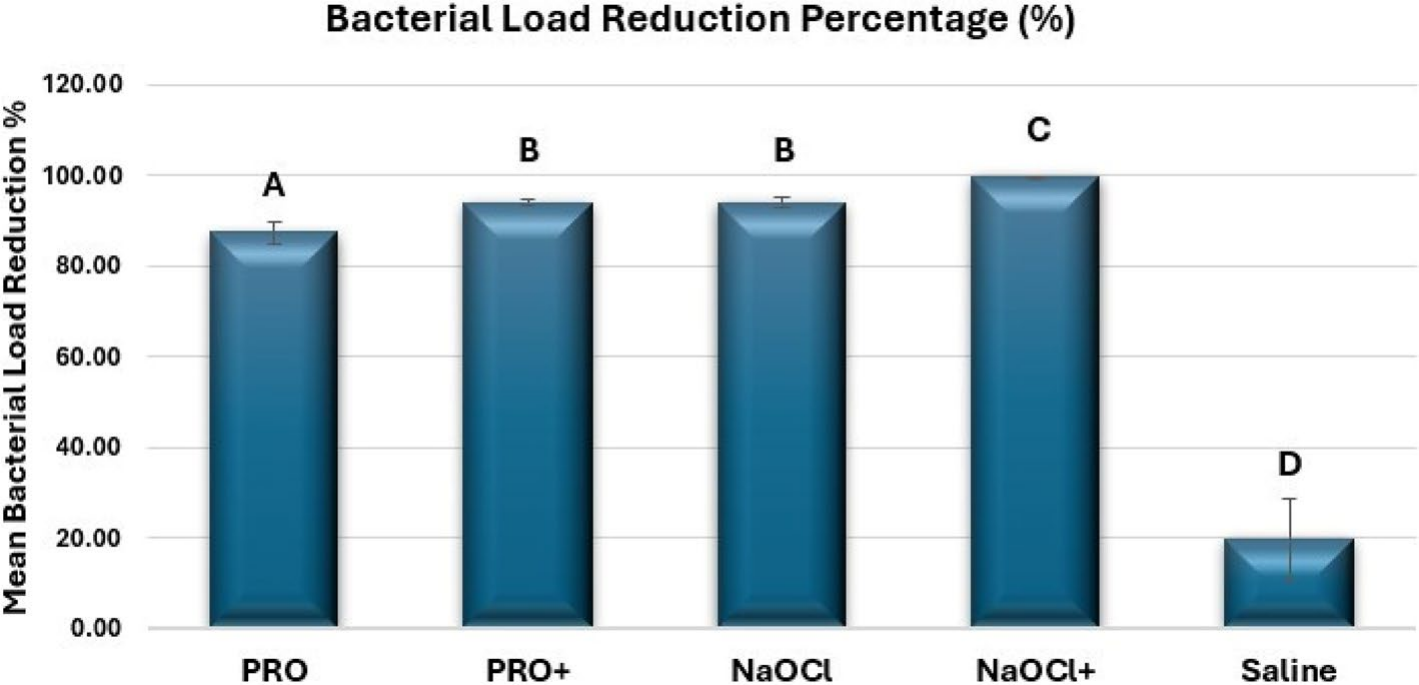
Bar chart showing mean bacterial load reduction percentages (%) in different groups. Different capital letters indicate statistically significant differences between the groups (*p* < 0.05)

Descriptive statistics of plate count (CFUs/mL) values and bacterial load reduction percentages for each of the tested groups are presented in Table 3 and Fig. 2.

**Table 3:** Descriptive statistics (mean ± standard deviation) for CFUs/mL of *E. faecalis* biofilm and bacterial load reduction percentage for different irrigation protocols

CFUs/mL (E. faecalis)				*Bacterial Load Reduction Percentage (%) *Mean ± SD
	**Pre-irrigation (S1) Mean ± SD**	**Post-irrigation (S2) Mean ± SD**	***p-value***	
**PRO**	531.00 ± 100.95^Y^	65.83 ± 5.91^Z^	< 0.001*	87.44 ± 1.25^A^
**PRO + **	515.00 ± 94.60^Y^	31.47 ± 5.84^Z^	< 0.001*	93.89 ± 0.38^B^
**NaOCl**	521.33 ± 104.62^Y^	30.83 ± 3.42^Z^	< 0.001*	94.01 ± 0.61^B^
**NaOCl + **	541.00 ± 94.82^Y^	1.60 ± 0.40^Z^	< 0.001*	99.70 ± 0.07^C^
**Saline**	531.67 ± 101.50^Y^	430.07 ± 103.51^Y^	0.831	19.65 ± 4.46^D^
***p-value***	1.000	< 0.001*		< 0.001*

Means with the same capital superscript have no significant difference at *p* < 0.05, * = Significant. *CFUs* Colony-forming units

Regarding the colony counting method, results indicated no significant difference in the pre-irrigation mean plate count (CFUs/mL) values of *E. faecalis* between the tested groups (*p-value* = 1.000), while a statistically significant difference was reported among them in post-irrigation values (*p-value* < 0.001). There was a statistically significant difference in the mean plate count (CFUs/mL) of *E. faecalis* between the pre-irrigation and post-irrigation values in each group, except for the saline group which showed no significant difference in the mean plate count between pre- and post-irrigation samples (*p-value* = 0.831).

All experimental groups exerted antibacterial activity against *E. faecalis* with a reduction in the mean plate count (CFUs/mL) values and an increase in the mean bacterial load reduction percentages. The lowest mean post-irrigation plate count (CFUs/mL) values were observed in NaOCl + group (1.60 ± 0.40), followed by NaOCl (30.83 ± 3.42), PRO + (31.47 ± 5.84), PRO (65.83 ± 5.91) and saline negative control (430.07 ± 103.51) groups respectively, corresponding to mean bacterial reduction percentages of (99.70 ± 0.07), (94.01 ± 0.61), (93.89 ± 0.38), (87.44 ± 1.25) and (19.65 ± 4.46) for each group respectively.

Statistically significant differences were observed among all groups (*p-value* < 0.001), except for the NaOCl and PRO + groups which did not exhibit any statistically significant difference (*p-value* = 1.000).

## Discussion

As persistent microorganisms inside the root canals are the main cause of treatment failure, there is a growing need for a potent antimicrobial agent that can target a wide range of endodontic pathogens while minimizing toxic and irritating side effects [2, 26]. Although there is evidence suggesting that some microorganisms could be beneficial, endodontic procedures have consistently emphasized their complete eradication from the root canal system. Up until now, little has been known about the possible application of probiotic microorganisms in endodontics. Therefore, this study was undertaken to assess the antibacterial effect of probiotics as a root canal irrigant for infected root canals containing biofilm-forming *E. faecalis*, aiming to introduce the novel approach of bacteriotherapy in endodontic therapy.

The *L. plantarum* probiotic strain was selected in our research as being commonly used in different endodontic studies [17, 20, 27]. *E. faecalis* is the most isolated microorganism in failed endodontically treated cases due to its ability to create biofilms that protect it from the host’s immune responses and antimicrobial treatments [4, 5]. So, the *E. faecalis* strain was chosen as the endodontic pathogen in the current study, being the predominant bacterium expressed in persistent infections [3]. A tooth model infected with 21 days old *E. faecalis* biofilm was employed in this study to simulate the clinical situation as per previously conducted studies [18, 28]. This specific age of biofilm was selected based on prior research indicating that biofilms of this age exhibit greater resistance and are harder to eradicate compared to those that are one or two weeks old [29].

Our study involved two stages: first, the assessment of MIC of the *L. plantarum* probiotic against *E. faecalis* through the agar well diffusion technique, which was further confirmed by the broth microdilution method which was found to be similar. Second was the CFUs/mL assessment to examine the antibacterial effect of ultrasonically activated probiotic irrigant compared to NaOCl. The MIC of *L. plantarum* probiotic against *E. faecalis* was found to be 25 mg/mL. So, we selected a 300 mg/mL concentration as it is higher than the MIC, aiming to enhance the antibacterial activity against the biofilm-forming *E. faecalis* within the tooth model.

Our results showed a significant bacterial load reduction of *E. faecalis* when the probiotic irrigant was used either activated or not in comparison to the control group and the pre-irrigation samples. However, NaOCl was significantly more effective, especially after ultrasonic activation. Consistent with our findings, Safadi et al. used the *L. plantarum* and *Lactobacillus casei* probiotic species and compared their antibacterial activity against *E. faecalis* to NaOCl irrigant and found that they exhibited superior efficacy in biofilm elimination and prevented its further regrowth [30]. Additionally, El-Sayed et al. revealed that *Lactobacillus rhamnosus* when used as an irrigant inhibited the *E. faecalis* growth in a tooth model [18]. Furthermore, several studies proved the antibacterial effectiveness of *L. plantarum* as an intracanal medicament against *E. faecalis* biofilm [20, 27, 28].

Our findings are supported by the nature of probiotics’ mechanism of action which involves preventing biofilm formation through competitive exclusion of pathogens and the production of bacteriocins and other antimicrobial compounds [31]. Also, Kim et al. proved that lipoteichoic acid released from *L. plantarum* microorganisms has an antibiofilm action that is effective against diseases induced by *E. faecalis* biofilm [32]. Similarly, Gupta affirmed in his study the role of probiotics in harmful pathogens elimination, pointing to mechanisms like the secretion of BLIS (bacteriocin-like inhibitory substances) as well as the modulation of local environmental pH level [33]. The probiotic irrigant used in our study was prepared by mixing lyophilized CFS of *L. plantarum* with sterile distilled water. The chemical composition of *L. plantarum* CFS has been studied by many authors using various techniques, documenting the presence of lactic acid (LA), hydrogen peroxide, and bacteriocins-peptides, which all have antimicrobial and/or antibiofilm activity [34]. Liang et al. investigated the antibiofilm properties of *L. plantarum* against *Streptococcus mutans*, a key contributor to dental caries [35]. Their findings revealed that the CFS of *L. plantarum* exhibited the strongest antibiofilm effect among the tested oral *Lactobacilli*. They also identified, through bioassay-guided isolation, the main antibiofilm substance, which was a mixture of LA and valine, with LA playing a dominant role as it significantly reduced exopolysaccharide production, resulting in a looser and thinner biofilm.

This study is the first of a kind to examine the effect of ultrasonically activated *L. plantarum* probiotic irrigant against *E. faecalis* biofilm over NaOCl. For this research, the Ultra X ultrasonic activation device was used that operates at 45 kHz working frequency. It is a lightweight cordless handpiece that is easy to use, offering two power settings, with the higher power option being utilized in this study.

According to our results, the ultrasonic activation of the probiotic irrigant significantly contributed to bacterial load reduction, resulting in an antibacterial effect similar to that of NaOCl irrigation. These findings can be explained by the fact that passive ultrasonic irrigation enhances the depth of irrigation penetration, reaching non-instrumented areas in the root canal system, aiding in the removal of leftover debris and bacteria by inducing cavitation and acoustic streaming inside the irrigant [36]. It also creates elevated shear stress in the root canal, leading to an overall decrease in the adhesion of bacterial biofilm compared to conventional syringe irrigation [37]. Energy transmitted through the ultrasonic waves causes strong disruptive action on bacterial biofilm through the acoustic streaming action [38]. According to Gambarini et al. in 2020, Ultra X was found to have significantly better effectiveness in cleaning accessory canals when used at maximum power compared to the sonic EdoActivator [39]. Also, the findings of a study conducted by Ada et al. were in partial agreement with our study where Ultra X reported the highest bacterial elimination results when compared to traditional syringe irrigation, manual dynamic activation, and sonic-powered irrigation [40].

The use of single-root-canalled teeth in the tooth model is regarded as a limitation for the current study, as it does not reflect the real clinical situation where the pulp anatomy is more complex and may result in different outcomes for the irrigant effectiveness against *E. faecalis*. Further clinical *in-vivo* studies are needed in the future with longer follow-up periods to reach a more accurate conclusion on the exact effect of probiotic irrigants on *E. faecalis* biofilm eradication.

## Conclusion

Within the limitation of the present study, ultrasonically activated *L. plantarum* probiotic irrigant revealed an antibacterial effect similar to that of the conventional NaOCl and can be used effectively to fight against *E. faecalis* biofilm. Our *in-vitro* study emphasized the benefits of shifting focus from the necessity of complete pathogen elimination to restoring the natural microbiome. The growth of harmful bacteria in infected root canals can be inhibited by probiotics, creating an environment where beneficial bacteria can thrive and promote the recovery of pulpal health.

## Data Availability

The datasets used and/or analyzed during the current study are available from the corresponding author on reasonable request.

## Abbreviations

ATCC: American Type Culture Collection
BHI: Brain heart infusion
BLIS: Bacteriocin-like inhibitory substances
CFS: Cell-free supernatant
CFUs/mL: Colony-forming units per milliliter
CLSI: Clinical Laboratory Standards Institute
EDTA: Ethylene-diamine-tetra-acetic acid
E. faecalis: Enterococcus faecalis
Hr: Hours
L. plantarum: Lactobacillus plantarum
LA: Lactic acid
MIC: Minimum inhibitory concentration
Mm: Millimeters
MRS: De Man, Rogosa, and Sharpe
NaOCl: Sodium hypochlorite
OD: Optical density
WL: Working length
ZOI: Zone of inhibition
